# Research priorities of endometriosis patients and supporters in Aotearoa New Zealand

**DOI:** 10.1111/ajo.13831

**Published:** 2024-05-05

**Authors:** Katherine Ellis, Rachael Wood

**Affiliations:** ^1^ Department of Chemical and Process Engineering University of Canterbury Christchurch New Zealand; ^2^ Endometriosis New Zealand Christchurch New Zealand; ^3^ Biomolecular Interaction Centre University of Canterbury Christchurch New Zealand

**Keywords:** chronic pain, endometriosis, New Zealand, patient priorities, research priorities

## Abstract

**Background:**

In New Zealand, an estimated 10% of women and people presumed female at birth have endometriosis, a disease characterised by the presence of tissue similar to the lining of the uterus, outside of the uterus.

**Aims:**

The purpose of this study was to characterise the research priorities of New Zealand endometriosis patients and their support networks in alignment with an Australian study. This will allow researchers to be able to ensure their research aligns with closing research gaps prioritised by those who directly experience the impacts of the disease.

**Methods and Materials:**

There were 1262 responses to an online Qualtrics survey advertised through Endometriosis New Zealand's social media accounts and mailing list to reach endometriosis patients and their support networks.

**Results:**

Overall, the highest research priorities for surgically or radiologically confirmed endometriosis patients, clinically suspected endometriosis patients, chronic pelvic pain patients, and their parents, partners, family members and friends were the management and treatment of endometriosis, followed by understanding endometriosis' cause, and improved capacity to diagnose endometriosis earlier. The key differences between the priorities of symptomatic participants and supporters were that symptomatic participants placed a significantly higher priority on understanding the cause of endometriosis, and supporters placed a significantly higher priority on improving the diagnosis of endometriosis.

**Conclusions:**

There is alignment between the ranking of general research priority areas for endometriosis in Australasia, allowing for clear priorities for future research teams to structure their work around patient‐centredness.

## INTRODUCTION

Characterised by the presence of endometrial‐like tissue outside of the uterus, endometriosis is a common, but heterogeneous and enigmatic, chronic health condition.^1^, ^2^ In New Zealand, endometriosis is expected to affect approximately 10% of women and people presumed female at birth.^3^ Within a longitudinal study, 7.8% of the 429 female participants had been diagnosed with endometriosis by age 38;^4^ however, it is unclear what the proportion of confirmed and clinically suspected diagnoses are a part of this value. Patients with surgically or radiologically confirmed^5^ endometriosis, with clinically suspected endometriosis, or chronic pelvic pain symptoms (CPP), are directly affected by negative impacts,^6^ exacerbated by the long‐term lack of international research funding into endometriosis.^7^ The estimated economic burden of endometriosis in Australia is $10.3 billion NZD annually.^8^ The population of Australia is five times larger than New Zealand, so if New Zealand's burden is similar per capita, then endometriosis care and productivity losses by patients cost the economy $2.05 billion NZD annually.

Prior assessments of research priorities in Australia, the UK, and Ireland, found the highest priorities were investigating the cause of endometriosis, disease management, non‐surgical diagnostic methods,^9^ establishing a cure, understanding the progression and spread, fertility maximisation and maintenance, and effective healthcare professional education.^10^ Comprehensive lists as part of the World Congress on Endometriosis have also been developed on the wide range of research endeavours and knowledge gaps that need to be addressed in endometriosis research.^11^ There are indications to suggest that patients and their support networks have different priorities to researchers, and medical practitioners who often dictate the course of research.^12^

In a New Zealand cohort of 50 patients, the top three priorities for changing the current status of endometriosis care in New Zealand were more subsidised care, more research funding, and improved educational resources in that order.^13^ The purpose of the current survey study was to assess the research priorities of those directly impacted by endometriosis, patients and their support networks in New Zealand. By making this information available, the opportunity will arise for research teams in New Zealand to ensure their work is aligned with the desires of those affected by the condition to channel their efforts to top priority concerns.

## MATERIALS AND METHODS

### Survey design

The survey utilised collected anonymous data regarding age, gender identity, ethnicity, region and rurality of residence, and delay to diagnosis. Participants identified whether they best identified as a surgically or radiologically confirmed endometriosis patient, a clinically suspected endometriosis patient, an individual with CPP, or whether they were a partner, parent, family member, or friend of an endometriosis patient.

Two ranking questions were incorporated into the survey, with one question ranking the five general research areas from a 2023 Australian study,^9^ and the second question using the 11 specific research priorities from the same study.^9^ Blinded to the research priorities listed in the ranking questions, participants organically shared their top research priority for endometriosis research in an open‐text question for separate collation and ranking. The design and distribution of the survey was approved by the Board of Governance of Endometriosis New Zealand, and ethics approval was obtained from the University of Canterbury Human Research Ethics Committee (Ref: HREC 2023/92‐LR/PS) following consultation with the Ngāi Tahu Consultation and Engagement Group.

### Participants and recruitment

The Qualtrics survey was open to participants from 26 September to 27 October 2023, with distribution primarily conducted through the platforms of Endometriosis New Zealand, including the use of their Facebook, Instagram, and LinkedIn platforms, as well as distributing the survey through their opt‐in mailing list. Social media posts were also made by members of the research team, and snowball sharing of social media posts was made by members of the public and interested organisations.

### Data and analysis

Statistical significance was set at *P* < 0.05. All statistical analyses were done using Qualtrics Stats iQ. Differences between groups in open‐ended research priorities were conducted using Pearson's χ^2^ tests. For the ranking questions, differences between age groups and gender identities were conducted using one‐way analysis of variance, and differences between symptomatic participants (confirmed diagnoses from surgery or radiology, clinically suspected diagnoses, CPP) and supporters (parents, partners, family members, friends), and between those with delays to diagnosis of less than ten years, or ten years or more, were conducted using ranked *t*‐tests. The grouping of ‘symptomatic’ was used to signify the individuals who presently or previously had experienced symptoms of endometriosis, relative to supporters. For this grouping it was assumed that to obtain a clinically suspected endometriosis diagnosis or exhibit CPP, the individual is likely to be, or have been symptomatic, while among the 1024 participants with self‐reported confirmed diagnoses, only three indicated they were asymptomatic to the point of diagnosis. This indicated that within the grouping termed ‘symptomatic’, as few as 0.26% had always been asymptomatic.

## RESULTS

### Participant demographics

There was a diverse range of participants (*n* = 1262) who reported surgically or radiologically confirmed endometriosis, clinically suspected endometriosis, or CPP, as well as parents, partners, family members and friends of endometriosis patients (Table 1). Participants were from all regions of New Zealand. Pasifika participants were associated with islands in the nations of the Cook Islands, Kiribati, Niue, Samoa, Tonga, and New Zealand. Māori participants were a part of 36 different iwi from all parts of the country, with 23.5% of Māori participants being a part of Ngāi Tahu/Kāi Tahu, a South Island iwi, 13.3% being a part of Ngāpuhi, a Northland iwi which is the largest in New Zealand, and 11.2% being a part of Ngāti Kahungunu, an iwi based on the North Island's east coast.

**Table 1:** Demographics of participants

	Total	Confirmed or symptomatic				Supporters			
		Surgically confirmed	Radiologically confirmed	Clinically suspected	CPP	Parent *N* = 34	Partner *N* = 29	Family *N* = 16	Friend *N* = 26
*N*	1262	955	69	107	26	105			
Age									
18–24	195 (15.5%)	128 (13.4%)	8 (11.6%)	40 (37.4%)	5 (19.2%)	14 (13.3%)			
25–34	507 (40.2%)	402 (42.1%)	23 (33.3%)	43 (40.2%)	10 (38.5%)	29 (27.6%)			
35–44	334 (26.5%)	265 (27.7%)	24 (34.8%)	18 (16.8%)	6 (23.1%)	21 (20.0%)			
45–54	170 (13.5%)	131 (13.7%)	13 (18.8%)	5 (4.7%)	4 (15.4%)	17 (16.2%)			
55+	54 (4.3%)	28 (2.9%)	1 (1.4%)	1 (0.9%)	1 (3.8%)	24 (22.9%)			
Gender identity									
Female	1197 (94.8%)	937 (98.1%)	67 (97.1%)	101 (94.4%)	24 (92.3%)	68 (64.8%)			
Male	36 (2.9%)	1 (0.1%)				35 (33.3%)			
Trans female	2 (0.2%)				1 (3.8%)	1 (1.0%)			
Trans male	2 (0.2%)	1 (0.1%)		1 (0.9%)					
Gender diverse	3 (0.2%)		1 (1.4%)	2 (1.9%)					
Non‐binary	19 (1.5%)	14 (1.5%)		3 (2.8%)	1 (3.8%)	1 (1.0%)			
Takatāpui	1 (0.1%)	1 (0.1%)							
Agender	1 (0.1%)		1 (1.4%)						
Ethnicity (multiple selections possible)									
NZ European	1073 (85.0%)	828 (86.7%)	51 (73.9%)	84 (78.5%)	21 (80.8%)	89 (84.8%)			
Māori	175 (13.9%)	140 (14.7%)	7 (10.1%)	15 (14.0%)	3 (11.5%)	10 (9.5%)			
Asian	34 (2.7%)	18 (1.9%)	5 (7.2%)	7 (6.5%)		4 (3.8%)			
Pacific Peoples	33 (2.6%)	25 (2.6%)	4 (5.8%)	1 (0.9%)	1 (3.8%)	2 (1.9%)			
Latin American	15 (1.2%)	8 (0.8%)	2 (2.9%)	3 (2.8%)		2 (1.9%)			
African	6 (0.5%)	5 (0.5%)		1 (0.9%)					
Middle Eastern	5 (0.4%)	5 (0.5%)							
Residence									
Rural	113 (9.0%)	81 (8.5%)	7 (10.1%)	12 (11.2%)	3 (11.5%)	10 (9.5%)			
Semi‐rural	159 (12.6%)	112 (11.7%)	14 (20.3%)	18 (16.8%)	3 (11.5%)	12 (11.4%)			
Urban	979 (77.6%)	755 (79.1%)	47 (68.1%)	76 (71.0%)	19 (73.1%)	82 (78.1%)			

### Research priorities

#### Organic top research priorities

**Figure 1:**
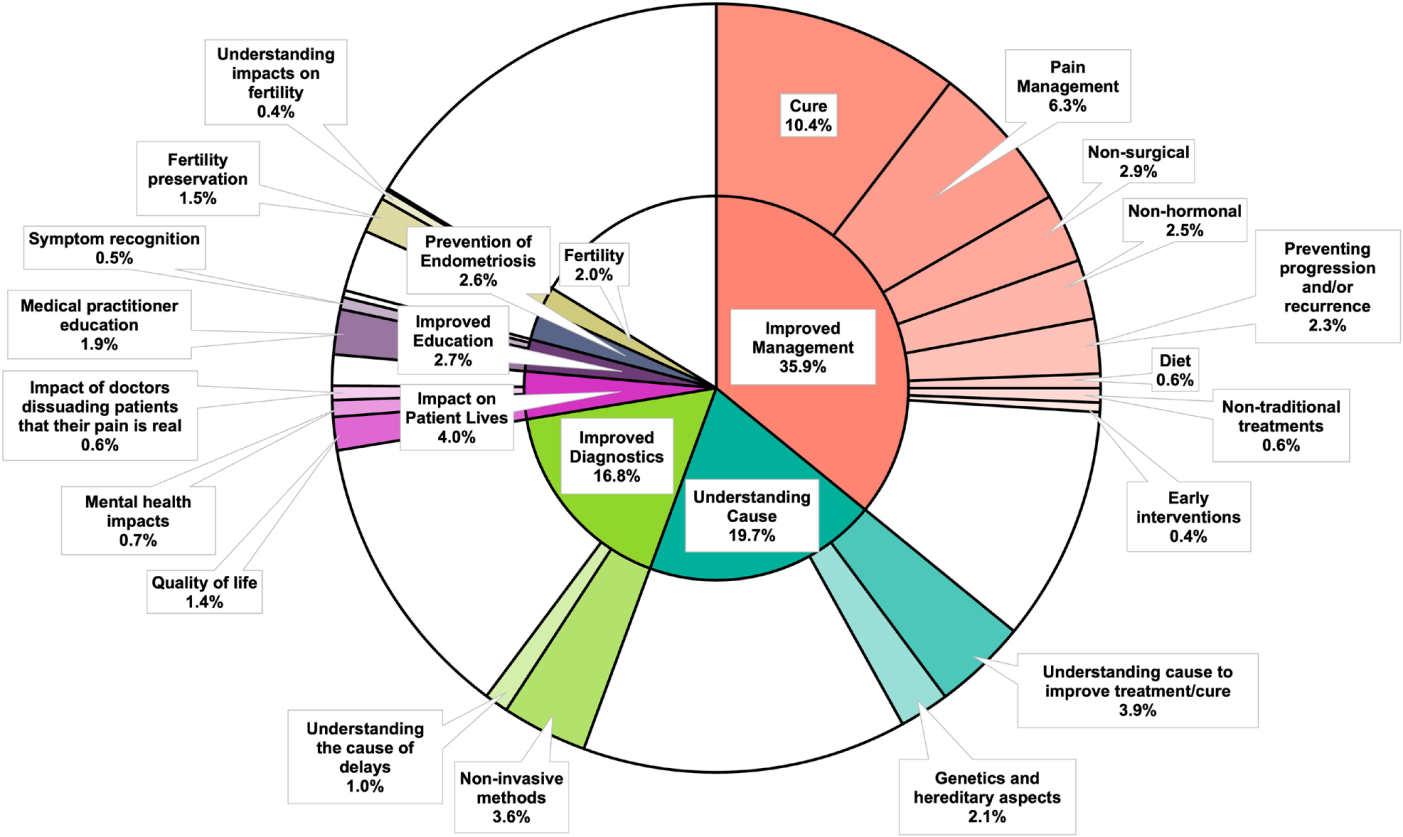
Plot of open‐ended top research priority results. Outer pie segments are sub‐topics of the inner pie segments. White sections are ‘other’ priorities or sub‐topics.

Before the ranking priority questions from the 2023 study by Armour et al,^9^ participants completed an open‐ended question where they organically highlighted their priority for endometriosis research. Each umbrella research priority had sub‐topic research priorities within them, with the most popular themes and sub‐topics summarised in Figure 1. The top priority was improved endometriosis management (35.9%) within which the top sub‐topics were a cure (2.0%) pain management (17.5%) and non‐surgical management (8.1%). This was followed by understanding the cause of endometriosis (19.7%) within which the most popular sub‐categories were an indication that understanding the cause would improve management options (19.8%) and research into the genetic nature of endometriosis (10.7%). The remaining top research priorities for participants were improved diagnostics (16.8%), impacts on patient lives (4.0%), improved education (2.7%), prevention (2.6%) and fertility research (2.0%).

Subsequent comparisons are between the stated group and other age groups, gender identities, or ethnicities, or between symptomatic and supporting participants, and between individuals with delays to diagnosis below ten years, or ten or more years. Within the symptomatic group, there were no significant differences between those reporting surgically confirmed, radiologically confirmed, or clinically suspected endometriosis, or those with CPP. Eighteen to 24‐year‐old participants were significantly more likely (*P* < 0.01) to rate improved endometriosis management as their top priority, while 45–54‐year‐old participants were significantly less likely (*P* < 0.05). Thirty‐five to 44‐year‐old participants were significantly more likely (*P* < 0.05) to consider preventing endometriosis their top priority. Eighteen to 24‐year‐old participants were also significantly less likely (*P* < 0.05) to have fertility as their top research priority, while participants 55+ and with delays of ten years or more were significantly more likely (*P* < 0.01) to have fertility as their top priority. Female and Māori participants (*P* < 0.05), and individuals with delays of less than ten years (*P* < 0.001) were significantly more likely to consider the cause of endometriosis their top research priority, while gender‐diverse participants were significantly less likely (*P* < 0.05). Male participants were significantly less likely to have the prevention of endometriosis as their top research priority (*P* < 0.05).

Symptomatic participants were significantly more likely (*P* < 0.01) to consider the cause of endometriosis their top priority and less likely to consider the improved diagnosis of endometriosis their top priority (*P* < 0.05), compared to supporters. Participants with delays to diagnosis of ten years or more were significantly more likely (*P* < 0.01) to consider improved diagnostics their top research priority. There were no significant differences with age, gender identity, delay to diagnosis, between supporters and symptomatic patients, or by ethnicity for the prioritisation of patient life impacts, or education.

#### Ranking of general research areas

**Figure 2:**
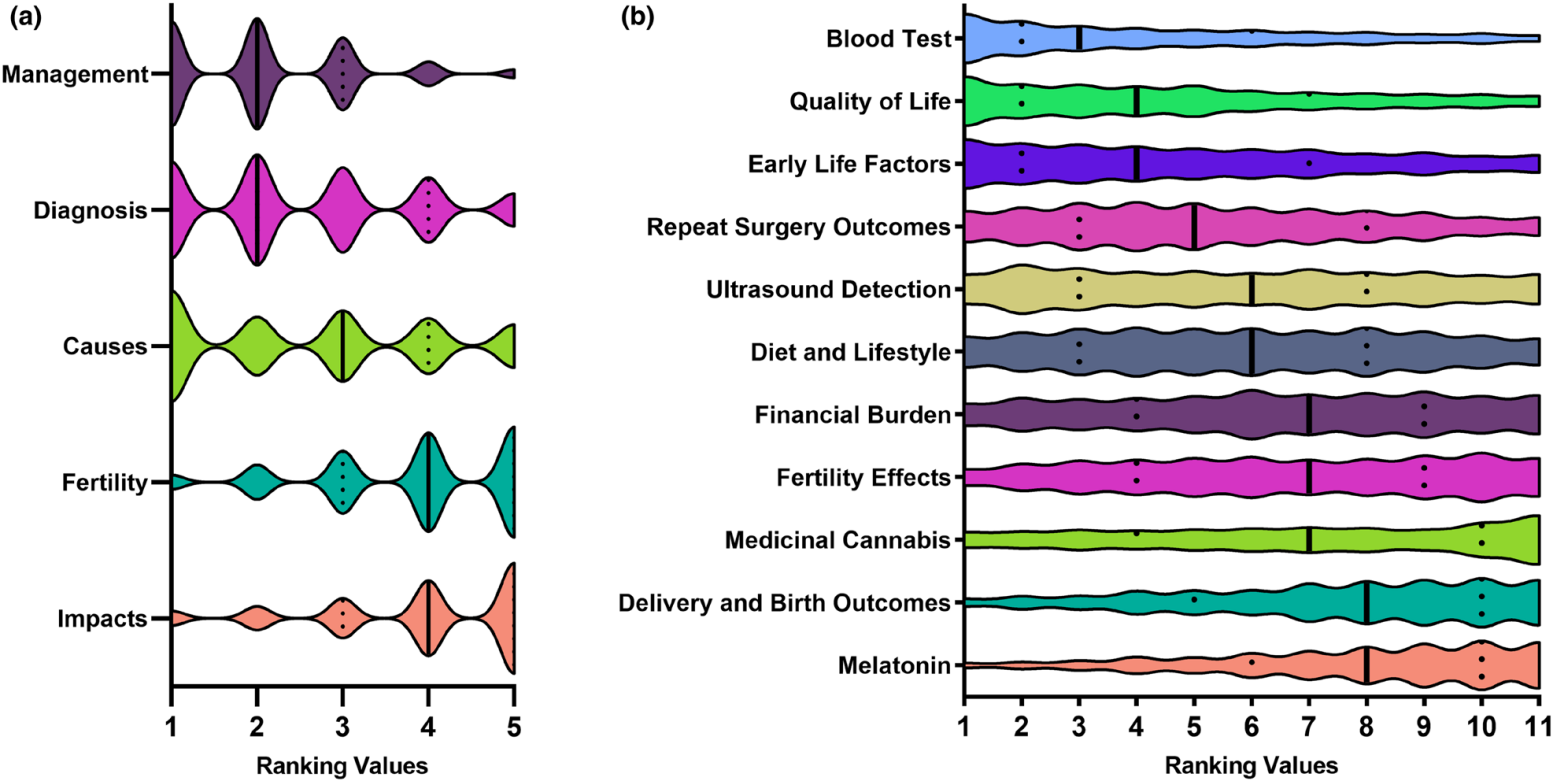
Violin plots showing the distribution of ranking votes for the (a) general research priorities where ‘1’ is top priority and ‘5’ is lowest priority and (b) specific research priorities showing median (black line) and interquartile range (black dotted lines) where ‘1’ is top priority and ‘11’ is lowest priority.

The results of the open‐ended question also align with the ranking of five general research areas (Fig. 2a, Table 2, 1 being highest priority, 5 being lowest priority) where the treatment and management of endometriosis symptoms (median score 2/5), the best ways of early diagnosis (2/5) and the causes of endometriosis (3/5) were ranked much higher than research into the fertility implications and impact of endometriosis on the individual and society (median score 4/5). The order of these results is the same general order as the 2023 Australian study by Armour et al.^9^

**Table 2:** Median and mean ranking values for general and specific research priorities

Overall ranking	General research priority *N* = 1262	Total median (1 highest priority, 5 lowest priority)	Total mean
1	What are the best ways of **treating** endometriosis and **managing** its symptoms?	2	2.13
2	What are the best ways of early **diagnosis** of endometriosis without surgery?	2	2.56
3	What are the **causes** of endometriosis?	3	2.59
4	What are the **fertility** implications of endometriosis?	4	3.80
5	How does endometriosis **impact** the individual and society?	4	3.93

Overall ranking	Specific research priority *N* = 1113	Total median (1 highest priority, 11 lowest priority)	Total mean
1	Is there a **blood test** that could screen for endometriosis early?	3	4.18
2	What is the **quality of life** and psychological impact of endometriosis?	4	4.59
3	What **early life factors** may predispose to endometriosis?	4	4.94
4	Does **repeat surgery** for endometriosis improve patient outcomes?	5	5.36
5	Can **ultrasound** reliably detect early endometriosis without surgery?	6	5.74
6	What is the effect of **diet and lifestyle** interventions (like engaging in regular exercise) on the symptoms of endometriosis?	6	5.86
7	What is the **financial burden** of living with endometriosis?	7	6.42
8	What is the **effect** of moderate–severe endometriosis on **fertility**?	7	6.58
9	How effective is using **medical cannabis** for endometriosis‐associated symptoms?	7	6.98
10	What is the impact of endometriosis on **delivery and birth** outcomes?	8	7.47
11	What is the efficacy of using **melatonin** for endometriosis‐associated symptoms?	8	7.87

The 18–24 age group rated the treatment and management of endometriosis priority significantly higher than every other age group (25–34, *P* = 0.0305, 35–44, *P* = 0.001, 45–54 *P* = 0.019, 55+, *P* = 0.024), as did participants with delays to diagnosis of ten years or more (*P* = 0.005). Meanwhile, the 45–54 age group rated fertility implications significantly lower than the 25–34 (*P* < 0.001) and 18–24 age groups (*P* = 0.0144). The 18–24 age group rated earlier diagnosis of endometriosis significantly lower than the 45–54 (*P* = 0.007) and 55+ (*P* = 0.046) age groups, and the 25–34 age group rated this priority significantly lower than the 45–54 age group (*P* = 0.011). Participants with confirmed diagnosis and delays of less than ten years rated the diagnosis priority significantly higher than those with delays of ten years or more (*P* = 0.003). There were no significant differences by age for the cause of endometriosis or the impact on the individual and society research priorities.

Female participants rated the fertility implications priority significantly higher than gender‐diverse participants (*P* < 0.001), while gender‐diverse participants rated the treatment and management of endometriosis symptoms significantly higher than female participants (*P* = 0.0121). There were no significant differences in priority ranking according to gender identity for the causes, impact, or earlier diagnosis of endometriosis research priorities.

There were two research priorities with significantly different rankings between symptomatic participants (confirmed diagnoses, clinically suspected endometriosis, or CPP) and supporting participants (parents, partners, family members, and friends). Supporters tended to consider the cause of endometriosis a lower research priority than symptomatic participants (*P* = 0.011), while symptomatic participants tended to consider the earlier diagnosis ranking priority to be a lower priority than supporters (*P* = 0.049). There were no significant differences in the rankings within the symptomatic grouping.

#### Ranking of specific priority areas

As with the Australian study,^9^ the ranking of the 11 specific priority areas (Fig. 2b, Table 2) did not necessarily align with associated general priority areas. Despite management being the top general research priority area in the open‐ended and prior ranking question, the specific research topics associated with management were ranked fourth (outcomes with repeat surgeries), sixth (diet and lifestyle interventions), ninth (medicinal cannabis) and 11th (melatonin). Meanwhile, research into diagnostic methods, which as a general area ranked third overall, ranked first (blood test) and fifth (non‐surgical diagnoses with ultrasound) in the specific research priorities, and the second overall specific priority, the quality of life and psychological impacts of endometriosis, is associated with the lowest rated general research area.

Early detection using ultrasound without surgery, tended to have higher priority among the 45–54 age group compared to the 18–24 (*P* < 0.001) and 25–34 age groups (*P* = 0.004), and among the 55+ age group compared to the 18–24 (*P* < 0.001) and 25–34 age groups (*P* = 0.002). This utilisation of ultrasound was also rated significantly higher by confirmed participants with delays of less than ten years compared to those with longer delays (*P* = 0.014). Having a blood test for endometriosis was ranked as less important for the 18–24 age group compared to the 35–44 (*P* = 0.010), 45–54 (*P* < 0.001) and 55+ age groups (*P* = 0.048), and less important in the 25–34 age group compared to the 45–54 age group (*P* = 0.004). The use of melatonin for endometriosis was ranked as significantly more important for the 35–44 age group compared to the 18–24 (*P* = 0.024) and 45–54 (*P* = 0.007) age groups. The effect of moderate–severe endometriosis on fertility was ranked as significantly more important among the 18–24 age group compared to the 35–44 age group (*P* = 0.032). The effect on delivery and birth outcomes was ranked as more important to the 25–34 age group than to the 45–54 age group (*P* = 0.046). All remaining priority areas had no significant differences between age groups.

Early non‐surgical detection using ultrasound was ranked as a lower priority by female participants than by male (*P* < 0.001) and gender‐diverse participants (*P* = 0.016). The impact of moderate–severe endometriosis on fertility was a lower priority for gender‐diverse participants than for male (*P* = 0.0016) and female participants (*P* < 0.001). Similarly, the effect on birth and delivery outcomes was a significantly lower priority for gender‐diverse than for female participants (*P* < 0.001). Male participants ranked the psychological impact of endometriosis significantly lower than female (*P* = 0.046) and gender‐diverse participants (*P* = 0.003). The financial burden of endometriosis was significantly lower ranked for male participants than for gender‐diverse participants (*P* = 0.024). There were no other significant differences in ranking according to gender identity.

Symptomatic participants ranked early detection without surgery by using ultrasound as a significantly lower priority than supporters (*P* < 0.001). Supporters ranked the psychological impact as a significantly lower priority than symptomatic participants (*P* = 0.015), along with the financial burden of endometriosis (*P* = 0.035). No other research priorities had significantly different rankings between symptomatic participants and supporters. Within the symptomatic grouping, those reporting a surgically confirmed diagnosis rated the priority of underlying the outcomes of repeat surgeries significantly higher than the participants with radiologically confirmed (*P* = 0.0101) and clinically suspected endometriosis (*P* = 0.0143), while surgically confirmed participants rated early detection with ultrasound as significantly less important than the radiologically confirmed participants (*P* = 0.0114).

Only one specific priority area was ranked with the same median value in this study as in the Australian counterpart,^9^ which is the early life factors that may predispose individuals to developing endometriosis. This study cohort has higher median values (lower prioritisation) for the outcomes of repeat surgeries, the reliability of ultrasound, the financial burden of endometriosis, the effect of moderate–severe endometriosis on fertility, and the effectiveness of medicinal cannabis. Conversely, this study has lower median values (higher prioritisation) of a blood test for endometriosis, the quality of life and psychological impacts, the effect of diet and lifestyle interventions, the impact on delivery and birth outcomes, and the use of melatonin for symptom management.

## DISCUSSION

### Limitations

#### Recall bias

Diagnostic delay data were based on participant self‐reports, so are subject to recall bias, and are unable to be validated with medical records as a result of anonymity, so the results of differences based on delay to diagnosis should be taken with caution. Furthermore, the delay to diagnosis data were collected from the point at which participants personally considered to have experienced ‘symptom onset’. This is complicated by the wide range of endometriosis symptoms.^14^ These symptoms can include the expected period pain, but more wide‐ranging symptoms such as gastro‐intestinal distress, neuro‐muscular issues, abnormal bleeding, fatigue, nausea, and infertility,^15^ which further exacerbate recall bias, particularly as the symptom(s) participants associated with symptom onset were not recorded.

#### Selection bias

Recruitment for this study was predominantly through the networks of the authors and importantly, Endometriosis New Zealand which is a patient organisation. Recruitment through patient organisations (*n* = 291) has been indicated as potentially producing cohorts with more affected relationships, and significantly lower physical quality of life in seven out of eight domains compared to the secondary care cohort (*n* = 63),^16^ which would likely influence the overall perspectives of the study cohort.

#### Completion rates

The second priorities ranking question had a drop‐off in response from the first ranking question (from 1262 respondents to 1113, an 11.8% decrease). This may relate to difficulties in re‐ordering the higher number of priorities (11 vs five) on devices such as smartphones; however, the number of priorities could not be reduced to avoid this issue while still maintaining consistency in the questioning with the 2023 Australian study.^9^ Additionally, due to the use of an anonymous link for all participants, it is impossible to know the overall response rate for the study, while the completion rate was 79.4% overall, and 90.1% of the organic and general priority questions which was the cut‐off for inclusion.

### Key findings

Our findings indicate three key things. First, in both organic open‐ended responses, and in the ranking of the five general priority areas, symptomatic participants and their supporters most readily supported the concept of research focusing on improvements to management and diagnosis, and in the development of a better understanding of the cause of endometriosis. There were trends of lower prioritisation of the early detection with ultrasound among symptomatic participants, particularly surgically confirmed endometriosis patients, which may relate to experiences with predominantly less sophisticated types of transvaginal ultrasounds which have been highlighted by patients in a qualitative study (*n* = 50) as particularly distressing.^15^, ^17^ This study also highlighted that while there were some differences between the ranking of specific research priorities between the New Zealand‐based cohort of this study, and the predominantly Australian‐based cohort of the 2023 Armour et al. study,^9^ there was strong alignment in the prioritisation of general priority areas. This highlights that an emphasis on research into the management, cause, and diagnosis of endometriosis is likely to be relatively universally accepted and appreciated by endometriosis patients and their supporters throughout New Zealand and Australia.

## Supporting information


**Data S1.** New Zealand endometriosis research priorities survey questions.
