# Supplementary material for: Research priorities of endometriosis patients and supporters in Aotearoa New Zealand
Source: Aust N Z J Obstet Gynaecol. 2024 May 5;64(6):548–55. doi: 10.1111/ajo.13831 (PMC11683752; doi:10.1111/ajo.13831)
Supplement: Supplementary file 1 — Data S1. New Zealand endometriosis research priorities survey questions. [file AJO-64-548-s001.pdf]

New Zealand Endometriosis Research Priorities Survey Questions:

1. What is your age?
    - 18-24
    - 25-34
    - 35-44
    - 45-54
    - 55+
    - Prefer not to say

2. To which gender identity do you most identify?
    - Female
    - Male
    - Transgender Female
    - Transgender Male
    - Gender Diverse
    - Non-binary
    - Other (Please Specify)
    - Prefer not to say

3. What is your ethnicity? (Select all that apply)
    - New Zealand European
    - Māori
    - Pacific Peoples
    - Asian
    - Middle Eastern
    - Latin American
    - African
    - Prefer not to say
    - Other (Please Specify)

4. (Only for Māori) Which iwi do you identify with? If you prefer not to say, please write N/A.

5. (Only for Pacific Peoples) Which Pacific Islands do you identify with? If you prefer not to say, please write N/A.

6. Which region of New Zealand do you live in?
    - Northland/Te Tai Tokerau
    - Auckland/Tāmaki-Makau-Rau
    - Bay of Plenty/Te Moana-a-Toi
    - Waikato
    - Taranaki
    - Gisborne/Te Tairāwhiti
    - Hawke's Bay/Te Matau-a-Māui
    - Manawatū-Whanganui

- Wellington/Te Whanga-nui-a-Tara
- Tasman/Te Tai-o-Aorere
- Nelson/Whakatū
- Marlborough/Te Tau Ihu-o-te-Waka
- West Coast/Te Tai Poutini
- Canterbury/Waitaha
- Otago/Ōtākou
- Southland/Murihiku
- Prefer not to say

7. Is where you live:
    - Rural
    - Semi-rural
    - Urban
    - Prefer not to say

8. Which best describes you?
    - I have a confirmed diagnosis of endometriosis from surgery
    - I have a confirmed diagnosis of endometriosis from ultrasound and/or MRI imaging
    - I have a suspected diagnosis of endometriosis from my doctor
    - I have chronic pelvic pain symptoms
    - I am the partner of an endometriosis patient
    - I am the parent of an endometriosis patient
    - I have an endometriosis patient in my family
    - I am the friend of an endometriosis patient

9. (Only for confirmed diagnosis of endometriosis) How long was it from when you started to have symptoms of endometriosis to when you received your diagnosis? (Open text)

10. What are 3-5 words you would use to describe the effects of endometriosis on you (or the effects on patients if you do not have endometriosis yourself)?

11. What is the number one thing about endometriosis you think should be researched? (Open text)

12. Please rank the following research areas from most to least important
    i. What are the best ways of early diagnosis of endometriosis without surgery?
    ii. What are the best ways of treating endometriosis and managing its symptoms?
    iii. How does endometriosis impact the individual and society?
    iv. What are the fertility implications of endometriosis?
    v. What are the causes of endometriosis?

13. Please rank the following research priorities from most to least important

i. Is there a blood test that could screen for endometriosis early?
ii. What is the impact of endometriosis on delivery and birth outcomes?
iii. What is the effect of diet and lifestyle interventions (like engaging in regular exercise) on the symptoms of endometriosis?
iv. How effective is using medical cannabis for endometriosis associated symptoms?
v. What is the quality of life and psychological impact of endometriosis?
vi. Does repeat surgery for endometriosis improve patient outcomes?
vii. What early life factors may predispose to endometriosis?
viii. What is the effect of moderate–severe endometriosis on fertility?
ix. What is the financial burden of living with endometriosis?
x. Can ultrasound reliably detect early endometriosis without surgery?
xi. What is the efficacy of using melatonin for endometriosis associated symptoms?
